# Knowledge of HIV transmission, prevention strategies and U = U among adult sexual and gender minorities in Brazil

**DOI:** 10.1002/jia2.26220

**Published:** 2024-02-20

**Authors:** Kayser Rogerio Oliveira Silva, Rayane Cupolillo Ferreira, Lara E. Coelho, Valdilea G. Veloso, Beatriz Grinsztejn, Thiago S. Torres, Paula M. Luz

**Affiliations:** ^1^ Escola Nacional de Saúde Pública Sergio Arouca, Fundação Oswaldo Cruz Rio de Janeiro Brazil; ^2^ Instituto Nacional de Infectologia Evandro Chagas, Fundação Oswaldo Cruz Rio de Janeiro Brazil

**Keywords:** HIV knowledge, Undetectable = Untransmittable (U = U), treatment‐as‐prevention, zero‐risk, sexual and gender minorities, Latin America

## Abstract

**Introduction:**

Although strong scientific evidence of the efficacy and effectiveness of treatment‐as‐prevention (TasP) is available, full endorsement of the “Undetectable = Untransmittable” (U = U) and “zero‐risk” messages could be improved. Increasing knowledge about HIV transmission, prevention and treatment is a critical component of care efforts. The study assessed knowledge of HIV transmission and prevention strategies, and the perceived accuracy of the slogan U = U among sexual and gender minorities (SGM) in Brazil.

**Methods:**

Cross‐sectional web‐based survey targeting adult SGM living in Brazil (2021−2022) recruited on social media and dating apps. We used the 12‐item HIV Knowledge Assessment (HIV‐KA) questionnaire to assess HIV knowledge, three items of which address pre‐exposure prophylaxis (PrEP), post‐exposure prophylaxis and TasP. Perceived accuracy of the U = U slogan was assessed with the question: “With regards to HIV‐positive individuals transmitting HIV through sexual contact, how accurate do you believe the slogan U = U is?”. We *a priori* grouped the study population into three mutually exclusive groups: people living with HIV (PLHIV), HIV negative and HIV unknown. We used logistic regression models to assess factors associated with high HIV knowledge and perception of the U = U as completely accurate.

**Results:**

Of 50,222 individuals accessing the questionnaire, 23,981 were included: 5071 (21.0%) PLHIV, 17,257 (71.5%) HIV negative and 1653 (6.9%) HIV unknown. The proportion of participants with high knowledge was significantly higher for PLHIV and HIV negative (48.1% and 45.5%, respectively) compared to 26.1% of HIV unknown. More PLHIV perceived U = U as completely accurate (80.4%), compared to 60.0% of HIV negative and 42.9% of HIV unknown. HIV knowledge correlates with perceived accuracy of the U = U slogan across all groups. Higher HIV knowledge was associated with higher income and education regardless of HIV status. Among HIV‐negative participants, PrEP awareness and use were associated with higher knowledge and accurate perception of the U = U slogan.

**Conclusions:**

Our findings show that HIV knowledge and perceived accuracy of U = U are strongly correlated, that knowledge differs according to HIV status, and that poor socio‐economic is linked to poor knowledge among SGM from Brazil. Educational strategies regarding TasP, U = U and zero risk targeting socio‐economically vulnerable populations are urgent in Brazil.

## 1 INTRODUCTION

Scientific evidence of the efficacy and effectiveness of treatment‐as‐prevention (TasP) has accrued from experimental [1] and large cohort studies [2, 3, 4] and were recently summarized in a systematic review that showed zero risk of sexual transmission of HIV with HIV viral load <1000 copies per ml [5]. Moreover, population‐level studies have shown that high coverage of antiretroviral therapy (ART) may be an effective approach to reduce HIV incidence [6]. The dissemination of TasP has been promoted since 2016 through the slogan “Undetectable = Untransmittable” (U = U) launched by the Prevention Access Campaign [7]. Additionally, the World Health Organization (WHO) released a policy brief at IAS 2023 (the 12th IAS Conference on HIV Science, in Brisbane, Australia), stating in a clear message that “people living with HIV who have an undetectable viral load using any WHO‐approved test and continue taking medication as prescribed have ‘zero risk’ of transmitting HIV to their sexual partner(s)” [8]. Beyond TasP, major advancements in HIV prevention include oral and injectable pre‐exposure prophylaxis (PrEP) [9, 10, 11].

Accurate knowledge about HIV transmission and the multiple prevention and treatment strategies is essential for people living with HIV (PLHIV), individuals at substantial risk of acquiring HIV, as well as the broader population. For PLHIV, accurate knowledge can empower them to make informed decisions about their health and take an active role in the uptake and adherence to ART [5, 12]. Indeed, a study conducted in 25 countries showed how PLHIV who discussed U = U with their healthcare provider reported improved health outcomes including adherence, viral suppression and sexual health [13]. For individuals at substantial risk of acquiring HIV, awareness of HIV risk and of the prevention strategies may foster health‐promoting attitudes and successful engagement in healthcare‐seeking behaviour [14]. Moreover, promoting accurate knowledge about HIV across the broader population reduces stigma and encourages HIV testing [15, 16].

Prior studies with gay, bisexual and other men who have sex with men (MSM) in Brazil found that only 37% [17], 24% [18] and 28% [19] of participants had high levels of HIV knowledge in 2009, 2016 and 2019, respectively. Given the disproportionately high burden of HIV among sexual and gender minorities (SGM) (10−30% HIV prevalence depending on year/population group [20, 21, 22]), it is important to monitor the level of knowledge about HIV transmission, prevention and treatment in these populations. This study assessed the level of knowledge of HIV transmission, prevention strategies and U = U among SGM. We also identified the socio‐demographic and behavioural factors associated with high levels of HIV knowledge and evaluated if high HIV knowledge was associated with perceived accuracy of the U = U slogan.

## 2 METHODS

### 2.1 Study design and population

Web‐based survey in Brazilian Portuguese was advertised using a geosocial networking (GSN) dating apps (Grindr, Hornet and Scruff) and social media (Facebook and Instagram). Requests for voluntary survey completion were sent through direct message inbox for Hornet, banners for Scruff and Grindr, and boosted posts for Facebook and Instagram, following approaches conducted in prior studies [23]. Individuals who met eligibility criteria (adults aged ≥18 years) and who acknowledged reading the informed consent text were directed to the questionnaire. Exclusion criteria included identifying as a cisgender woman, self‐report of completing the questionnaire previously or duplicated internet protocol (I.P.) address (first occurrence was kept) and living abroad. Participants were not required to self‐identify as a SGM.

The study's sample was a convenience sample that targeted the maximum number of participants possible without a specific sample size calculation. This study was approved by the local ethics committee (#CAAE 01777918.0.0000.5262 and #CAAE 82021918.0.0000.5262). No incentives were provided for completing the survey and no personally identifiable information was collected. I.P. addresses were only used to apply the exclusion criterion mentioned above.

### 2.2 Study instrument

The survey instrument was programmed on Alchemer® and remained open for completion from May 2021 to January 2022. Four authors systematically checked the usability and technical functionality of the electronic questionnaire in different platforms and operating systems before launching the study.

For this analysis, we considered three sections of the questionnaire. Section 1 included items on socio‐demographic information (age, gender, sexual orientation, race, education level, family monthly income, state of residence and steady partner). Section 2 included items referring to prior HIV testing and HIV test results and subsequent questions differed based on HIV status. HIV‐negative and HIV‐unknown participants were questioned about PrEP awareness, PrEP use (current, never or past) and sexual behaviour in the past 6 months (number of sexual partners and condomless receptive anal sex). PLHIV were questioned about the use of ART, and if in use, adherence in the past 7 days was measured using the WebAd‐Q instrument [24]. This instrument includes three questions that should be answered as Yes, No or I don't remember, with individuals being classified as adherent (yes vs. no) if they answer “No” to all three questions.

Section 3 included items of the HIV knowledge assessment tool (HIV‐KA), a 12‐item measure previously validated by our team using the same study design and target population as in this study [25] and previously used to assess knowledge among MSM in Brazil [18]. Items address aspects of HIV transmission, prevention or treatment (all items provided in Table S1). Three items were of particular interest to this study: item #1 that focuses on PrEP; item #2 that focuses on TasP: and item #4 that focuses on post‐exposure prophylaxis (PEP). The response format of all items included the options “true,” “false” and “I don't know.” The total score of the instrument was calculated by summing across all items that the participant answered correctly (“I don't know” was coded as an incorrect response) with higher scores reflecting greater HIV knowledge. Of note, to minimize participant burden, a fraction (*n* = 2065, 41%) of PLHIV were not presented with the items of the HIV‐KA. When comparing the socio‐demographic characteristics of the participants who were presented with the items versus not, we found no differences between groups (all chi‐squared tests *p*‐values >0.05) regarding age, gender or sexual orientation, though participants who were presented with the HIV‐KA had lower income (17% vs. 8% had <1 minimum wage) and lower education level (40% vs. 27% had up to secondary education).

Perceived accuracy of the U = U slogan was assessed with the question: “With regards to HIV‐positive individuals transmitting HIV through sexual contact, how accurate do you believe the slogan U = U is?” as used in previous studies [26, 27], though we added an explanation in our translated version stating “meaning that people who have HIV but have undetectable viral load do not transmit HIV through sex.” Response options were based on a 4‐point Likert‐type scale from “Completely accurate” to “Completely inaccurate” plus a fifth option (I don't know what “undetectable” means).

### 2.3 Statistical analysis

Participants’ answers to the HIV testing and HIV status items allowed us to group the study population into three mutually exclusive groups: PLHIV, participants who reported previously testing for HIV and having a negative test result (HIV negative), and participants reporting never having tested (HIV unknown). The rationale for this *a priori* categorization was that prior studies [26, 28] showed that perceived accuracy of U = U differs significantly by HIV status with PLHIV showing greater perceived accuracy, whereas individuals who are unaware of their HIV status have the lowest perceived accuracy.

Participants’ characteristics were described overall and according to each study group (PLHIV, HIV negative, HIV unknown). Following, we described the perceived accuracy of U = U, HIV knowledge, awareness of PrEP, use of PrEP, ART use and adherence, as applicable, for each study group. Beyond describing HIV knowledge scores, we also standardized the score to facilitate comparisons with previous studies. First, we recalculated the scores using a scale from 0 to 10 (divided participant's score by 12 and multiplied by 10). Then, using the population score distribution, we took the values of the 75th percentile as a cutoff point for creating a dichotomous variable: low/moderate knowledge (<75th percentile) and high knowledge (≥75th percentile). We used chi‐square tests to compare, across groups, the proportions of participants endorsing each of the five response options of the perceived accuracy of the U = U item. Then, we assessed the correlation between variables indicative of greater knowledge. Among HIV‐negative participants, we assessed whether knowledge was correlated with the use of PrEP and, among PLHIV, whether it was associated with ART adherence.

Finally, logistic regression models were used to assess the factors associated with knowledge (high knowledge vs. low/moderate knowledge) and perceived accuracy of U = U (completely accurate vs. partially accurate/inaccurate or completely inaccurate; those reporting not knowing what undetectable meant were removed) by study group. All potentially relevant variables, as applicable by study group, were included *a priori* in one adjusted model. Models were also adjusted for geographic region, month of participation and recruitment strategy to account for heterogeneities that might have been introduced by these variables [29]. HIV knowledge was included as a potential predictor of perceived accuracy of U = U. Analyses were performed using R (The R project www.r‐project.org, version 4.2.0).

## 3 RESULTS

### 3.1 Study population

**Figure 1:**
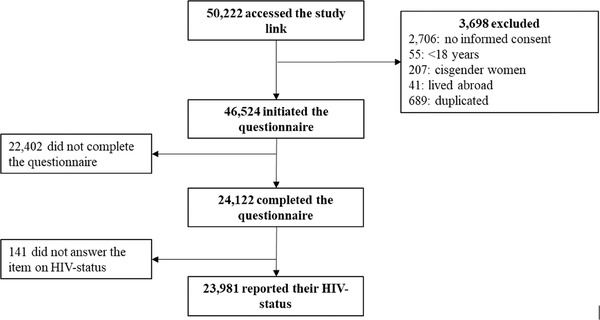
Flowchart of study inclusion and exclusion criteria.

From May 2021 to January 2022, a total of 50,222 individuals accessed the questionnaire, 3689 were excluded: 2761 were deemed ineligible (did not provide informed consent or were <18 years old) and 937 met exclusion criteria (Figure 1). From the 46,524 participants who initiated the questionnaire, 22,402 (48.15%) did not finish it. Among those who completed the questionnaire (*n* = 24,122), recruitment was highest through GSN dating apps, with 10,246 (42.5%), 5460 (22.6%) and 2129 (8.8%) of the participants reporting access to the study through Hornet, Grindr and Scruff, respectively. Recruitment through different social media was as follows: 3043 (12.6%), 2482 (10.3%) and 386 (1.6%) through Facebook, Instagram and WhatsApp, respectively. The remaining 376 individuals (1.6%) reported other recruitment channels.

Out of the 24,122 participants who completed the questionnaire, 23,981 (99.4%) answered the HIV testing and HIV status items and were included in this analysis: 5071 (21.0%) were PLHIV, 17,257 (71.5%) were HIV negative and 1653 (6.9%) were HIV unknown (Table 1). Mean age was highest among PLHIV (38.6 years) and lowest among HIV unknown (31.9 years). Sexual orientation differed by group, among PLHIV, 88.6% were gay and 9.4% were bisexual, the corresponding percentages were 84.0% and 12.7% for HIV negative and 72.4% and 18.0% for HIV unknown. Income and education were similar across the three groups with HIV‐unknown participants tending towards lower income and lower education. Compared to HIV unknown, the HIV‐negative group reported more sexual partners (>10 partners: 22.9% vs. 10.7%) and more frequent condomless receptive anal sex (49.0% vs. 41.6%).

**Table 1:** Participant's characteristics according to reported HIV status: people living with HIV (PLHIV), HIV negative and HIV unknown, Brazil, 2021 (*n* = 23,981)

	PLHIV	HIV negative	HIV unknown
Participants	5071	17,257	1653
**Age**			
Mean (SD)	38.6 (9.9)	35.4 (9.7)	31.9 (10.9)
Median (IQR)	38 (31−45)	34 (28−41)	29 (23−38)
18−24 years, *n* (%)	266 (5.2)	1850 (10.7)	486 (29.4)
25−29 years, *n* (%)	695 (13.7)	3562 (20.6)	346 (20.9)
30−34 years, *n* (%)	970 (19.1)	3628 (21.0)	274 (16.6)
35−44 years, *n* (%)	1844 (36.4)	5317 (30.8)	325 (19.7)
≥45 years, *n* (%)	1296 (25.6)	2900 (16.8)	222 (13.4)
**Gender**			
Cisgender man	4973 (98.1)	16,880 (97.8)	1602 (96.9)
Transgender man	5 (0.1)	31 (0.2)	5 (0.3)
Transgender woman	10 (0.2)	42 (0.2)	6 (0.4)
*Travesti*	5 (0.1)	15 (0.1)	4 (0.2)
Non‐binary	78 (1.5)	289 (1.7)	36 (2.2)
**Sexual orientation**			
Gay	4491 (88.6)	14,503 (84)	1196 (72.4)
Bisexual	478 (9.4)	2191 (12.7)	312 (18.9)
Heterosexual	43 (0.8)	299 (1.7)	111 (6.7)
Other	59 (1.2)	264 (1.5)	34 (2.1)
**Race**			
White	2850 (56.5)	10,104 (58.9)	909 (55.4)
*Pardo*/mixed	1480 (29.3)	4771 (27.8)	490 (29.8)
Black	609 (12.1)	1940 (11.3)	206 (12.5)
Asian	44 (0.9)	209 (1.2)	22 (1.3)
Indigenous	60 (1.2)	140 (0.8)	15 (0.9)
**Family Monthly income^a^**			
No income	253 (5.0)	818 (4.8)	85 (5.2)
< 1 minimum wage	415 (8.3)	1212 (7.1)	188 (11.6)
1−2 minimum wage	856 (17.1)	2568 (15.1)	331 (20.4)
2−3 minimum wage	793 (15.8)	2527 (14.8)	283 (17.4)
3−4 minimum wage	606 (12.1)	2021 (11.9)	209 (12.9)
4−6 minimum wage	773 (15.4)	2591 (15.2)	206 (12.7)
6−10 minimum wage	735 (14.6)	2720 (16)	183 (11.3)
>10 minimum wage	588 (11.7)	2571 (15.1)	140 (8.6)
**Education level**			
None	1 (0)	3 (0)	0 (0)
Incomplete basic	71 (1.4)	101 (0.6)	23 (1.4)
Basic	203 (4.0)	398 (2.3)	79 (4.8)
Secondary	1467 (29.0)	4373 (25.4)	686 (41.7)
Tertiary	1875 (37.1)	6715 (39.0)	565 (34.3)
Post‐tertiary	1442 (28.5)	5643 (32.7)	293 (17.8)
**Region**			
North	130 (2.6)	401 (2.3)	37 (2.2)
Northeast	456 (9)	2038 (11.8)	224 (13.6)
Central‐west	343 (6.8)	1257 (7.3)	95 (5.7)
Southeast	3423 (67.5)	11,398 (66)	1104 (66.8)
South	719 (14.2)	2163 (12.5)	193 (11.7)
**Live in capital/metropolitan region**			
No	1284 (25.3)	4889 (28.3)	658 (39.8)
Yes	3787 (74.7)	12,368 (71.7)	995 (60.2)
**Have a steady partner**			
No	3581 (70.6)	12,080 (70)	1245 (75.3)
Yes	1490 (29.4)	5177 (30)	408 (24.7)
**How many sexual partners in P6M**			
None		1349 (7.8)	338 (20.6)
1−5		8950 (52.1)	932 (56.9)
6−10		2948 (17.2)	193 (11.8)
>10		3942 (22.9)	176 (10.7)
**Had condomless receptive anal sex**			
No		8768 (51)	960 (58.4)
Yes		8425 (49)	685 (41.6)
**When was your last HIV test**			
Never tested			
<3 months ago		6778 (39.3)	
3−6 months ago		3152 (18.3)	
6−12 months ago		2965 (17.2)	
>1 year ago		4362 (25.3)	

Abbreviations: HIV, human immunodeficiency virus; IQR, interquartile range; PLHIV, people living with HIV; P6M, past 6 months; SD, standard deviation.

^a^One minimum wage corresponded to BRL 1212 (~USD 242) in 2022.

### 3.2 HIV knowledge and perceived accuracy of U = U

Though the mean HIV‐KA score did not differ by group (Table 2), the proportion of participants with high knowledge was significantly higher for PLHIV and HIV‐negative participants (48.1% and 45.5%, respectively) compared to 26.1% of HIV unknown. When looking at the three items of interest, items #1 (PrEP), #2 (TasP) and #4 (PEP), >85% of PLHIV and HIV‐negative participants answered these items correctly compared with ≤80% of HIV‐unknown participants. Across all three groups, PEP knowledge was highest, whereas PrEP knowledge was lowest, except for the HIV‐negative group. PrEP awareness was also higher for the HIV‐negative group (94.9%) than the HIV‐unknown group (82.8%). Among PLHIV, 96.7% were on ART and 57.3% were classified as adherent in the past 7 days.

**Table 2:** Participants’ perception of the slogan U = U, HIV knowledge, awareness of PrEP, PrEP use, and ART use and adherence according to reported HIV status: people living with HIV (PLHIV), HIV negative and HIV unknown, Brazil, 2021 (*n* = 23,981)

	PLHIV (*n*; %)	HIV negative (*n*; %)	HIV unknown (*n*; %)
**Participants**	5071	17,257	1653
**HIV knowledge measured by HIV‐KA**	*n* = 3006^a^		
Mean score (SD)	11.1 (1.2)	11 (1.4)	10.1 (1.9)
High knowledge	1447 (48.1)	7859 (45.5)	432 (26.1)
PrEP (item #1) correct	2658 (88.4)	14,791 (85.7)	1164 (70.4)
TasP (item #2) correct	2796 (93.0)	14,588 (84.5)	1176 (71.1)
PEP (item #4) correct	2850 (94.8)	16,169 (93.7)	1338 (80.9)
**Awareness of PrEP**			
No		883 (5.1)	285 (17.2)
Yes		16,374 (94.9)	1368 (82.8)
**PrEP use**			
Never		13,510 (81)	
Currently using		2302 (13.8)	
Used in the past		873 (5.2)	
**Use of ART**			
No	168 (3.3)		
Yes	4892 (96.7)		
**Adherence measured by WebAd‐Q** ^b^			
No	2087 (42.7)		
Yes	2806 (57.3)		

Abbreviations: ART, antiretroviral therapy; HIV, human immunodeficiency virus; PEP, post‐exposure prophylaxis; PLHIV, people living with HIV; PrEP, pre‐exposure prophylaxis; TasP, treatment as prevention.

PrEP item (item #1): “There are medications for HIV‐negative people to take before having sex with other people to prevent HIV infection.”

TasP item (item #2): “An HIV‐infected person who is taking HIV/AIDS medications has a lower risk of transmitting the virus to another person.”

PEP item (item #4): “There are medications for HIV/AIDS to be used after a situation of risk of infection (i.e. unprotected sex, sexual violence, etc).”

^a^ In one application of the questionnaire, participants who reported living with HIV (*n* = 2065) were not asked to answer the 12 HIV knowledge items and thus the denominator for this variable is 3006.

^b^ Adherence was defined as a “No” response to all three adherence items as per WebAd‐Q instrument.

**Figure 2:**
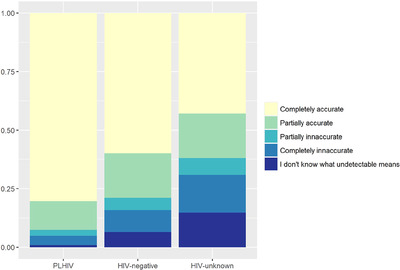
Proportion of participants who rated the item “With regards to HIV‐positive individuals transmitting HIV through sexual contact, how accurate do you believe the slogan U = U is?” as Completely inaccurate to 4 Completely accurate, or who said they did not know what “undetectable” meant by study group: people living with HIV (PLHIV), HIV‐negative participants and participants who did not know their HIV status (HIV unknown).

More PLHIV perceived U = U as completely accurate (80.4%), compared to 60.0% of HIV negative and 42.9% of HIV unknown (Figure 2, chi‐square test *p*‐value <0.001). Conversely, the fraction of individuals reporting “not knowing what undetectable is” also differed by group; only 0.9% of PLHIV selected this response compared to 6.5% of HIV negative and 14.8% of HIV unknown (chi‐square test *p*‐value <0.001). Overall, high knowledge correlates with perceived accuracy of the U = U slogan as completely accurate (Table 3). For all groups, HIV knowledge scores were highest among those who perceived U = U as completely accurate and lowest among those who perceive it as completely inaccurate. For PLHIV, among those who answered items #1, #2 and #4 correctly, ∼80% said that U = U was completely accurate. For the HIV‐negative group, these percentages were closer to 70%. For the HIV‐unknown group, among those who answered the items correctly, ∼55% said that the slogan U = U was completely accurate.

**Table 3:** Participants’ perception of the accuracy of the U = U slogan as a function of their knowledge, by study group: people living with HIV (PLHIV), HIV negative and HIV unknown, Brazil, 2021

	Perceived accuracy of the U = U slogan			
	Completely accurate	Accurate	Inaccurate	Completely inaccurate
**PLHIV**				
HIV knowledge score				
Mean (SD)	11.2 (1.0)	10.9 (1.4)	10.6 (1.5)	10.3 (1.7)
High knowledge (n; %)	1199 (83.0)	186 (12.9)	26 (1.8)	33 (2.3)
PrEP (item #1) correct (n; %)	2108 (79.9)	380 (14.4)	64 (2.4)	86 (3.3)
TasP (item #2) correct (n; %)	2196 (79.1)	428 (15.4)	73 (2.6)	80 (2.9)
PEP (item #4) correct (n; %)	2215 (78.4)	437 (15.5)	74 (2.6)	101 (3.6)
**HIV negative**				
HIV knowledge score				
Mean (SD)	11.3 (1.0)	11 (1.3)	10.6 (1.5)	10.3 (1.6)
High knowledge (n; %)	5579 (72.4)	1395 (18.1)	305 (4)	422 (5.5)
PrEP (item #1) correct (n; %)	9480 (67.2)	2792 (19.8)	708 (5)	1118 (7.9)
TasP (item #2) correct (n; %)	9628 (68.7)	2825 (20.2)	670 (4.8)	895 (6.4)
PEP (item #4) correct (n; %)	10,062 (65.7)	3084 (20.1)	797 (5.2)	1372 (9)
**HIV unknown**				
HIV knowledge score				
Mean (SD)	10.7 (1.5)	10.4 (1.7)	10 (1.6)	9.4 (2.1)
High knowledge (n; %)	254 (62.0)	97 (23.7)	21 (5.1)	38 (9.3)
PrEP (item #1) correct (n; %)	576 (55.4)	244 (23.5)	76 (7.3)	144 (13.8)
TasP (item #2) correct (n; %)	614 (57.2)	261 (24.3)	77 (7.2)	121 (11.3)
PEP (item #4) correct (n; %)	636 (53.9)	266 (22.5)	91 (7.7)	188 (15.9)

Abbreviations: HIV, human immunodeficiency virus; PEP, post‐exposure prophylaxis; PLHIV, people living with HIV; PrEP, pre‐exposure prophylaxis; TasP, treatment as prevention; U = U, undetectable equals untransmittable.

PrEP item (item #1): “There are medications for HIV‐negative people to take before having sex with other people to prevent HIV infection.”

TasP item (item #2): “An HIV‐infected person who is taking HIV/AIDS medications has a lower risk of transmitting the virus to another person.”

PEP item (item #4): “There are medications for HIV/AIDS to be used after a situation of risk of infection (i.e. unprotected sex, sexual violence, etc).”

### 3.3 Predictors of high HIV knowledge

For PLHIV, income and education level were the strongest predictors of high HIV knowledge with almost double the odds of high knowledge among those at the highest level of income and education (Table 4). Reporting ART adherence in the past 7 days was also associated with high HIV knowledge among PLHIV. Among HIV‐negative participants, both younger and older individuals (compared to those aged 30−34 years) had lower odds of high HIV knowledge. Self‐report of sexual orientation as gay (vs. other), higher income and higher education level were associated with high HIV knowledge among HIV‐negative participants. Additionally, an increased number of sexual partners, PrEP awareness and current/past PrEP use were associated with high HIV knowledge, whereas having been tested for HIV ≥3 months ago (compared to <3 months ago) was associated with lower odds of high HIV knowledge.

**Table 4:** Factors associated with high HIV knowledge by study group (people living with HIV [PLHIV], HIV negative and HIV unknown), Brazil, 2021 (*N* = 20,699)

	PLHIV (*n* = 2865)		HIV negative (*n* = 16,244)		HIV unknown (*n* = 1590)	
	High knowledge *N* (%)	aOR (95% CI)	High knowledge *N* (%)	aOR (95% CI)	High knowledge *N* (%)	aOR (95% CI)
**Age (years)**						
18−24	76 (40)	1.3 (0.9−1.87)	674 (36.4)	0.82 (0.72−0.93)	97 (20)	1.01 (0.68−1.5)
25−29	202 (43.6)	1.07 (0.82−1.38)	1527 (42.9)	0.85 (0.77−0.94)	107 (30.9)	1.42 (0.97−2.07)
30−34	281 (46.4)	Ref.	1789 (49.3)	Ref.	72 (26.3)	Ref.
35−44	545 (51.3)	1.13 (0.92−1.4)	2613 (49.1)	0.99 (0.9−1.08)	92 (28.3)	1.16 (0.79−1.72)
45 or more	343 (50)	1.03 (0.81−1.3)	1256 (43.3)	0.82 (0.74−0.91)	64 (28.8)	1.19 (0.77−1.83)
**Gender**						
Cisgender man	1429 (48.4)	1.69 (0.92−3.12)	7729 (45.8)	1.15 (0.91−1.46)	421 (26.3)	1.16 (0.53−2.53)
Other	18 (32.7)	Ref.	130 (34.5)	Ref.	11 (21.6)	Ref.
**Sexual orientation**						
Gay	1297 (49)	0.73 (0.42−1.26)	6821 (47)	1.41 (1.14−1.74)	346 (28.9)	2.11 (1.15−3.87)
Bisexual	120 (40)	0.6 (0.33−1.08)	871 (39.8)	1.23 (0.98−1.55)	66 (21.2)	1.53 (0.79−2.95)
Other	30 (50)	Ref.	167 (29.7)	Ref.	20 (13.8)	Ref.
**Race**						
White	880 (51.7)	Ref.	4926 (47.8)	Ref.	262 (28.1)	Ref.
Pardo	415 (43.7)	0.88 (0.74−1.05)	2067 (42.1)	0.88 (0.82−0.95)	121 (24)	0.88 (0.67−1.17)
Black	152 (42.9)	0.87 (0.67−1.12)	839 (43.2)	0.96 (0.87−1.07)	45 (21.8)	0.76 (0.52−1.13)
**Family Monthly Income** ^a^						
<1 minimum wage	157 (31.2)	Ref.	652 (32.1)	Ref.	43 (15.8)	Ref.
1−3 minimum wage	436 (43.7)	1.54 (1.21−1.95)	1975 (38.8)	1.2 (1.07−1.34)	156 (25.4)	1.72 (1.16−2.55)
3−6 minimum wage	412 (53.5)	1.96 (1.51−2.54)	2232 (48.4)	1.53 (1.36−1.72)	114 (27.5)	1.60 (1.05−2.45)
>6 minimum wage	442 (60.1)	2.2 (1.67−2.91)	2909 (55)	1.75 (1.55−1.98)	113 (35)	1.93 (1.23−3.01)
**Education**						
Up to secondary	443 (37.4)	Ref.	1657 (34)	Ref.	153 (19.4)	Ref.
Tertiary	554 (51.2)	1.47 (1.22−1.77)	3090 (46)	1.29 (1.19−1.41)	168 (29.7)	1.45 (1.09−1.94)
Post‐tertiary	450 (61.1)	1.92 (1.53−2.41)	3108 (55.1)	1.63 (1.48−1.79)	109 (37.2)	1.84 (1.28−2.65)
**Live in capital/metropolitan region**						
No	386 (49.2)	Ref.	2171 (44.4)	Ref.	162 (24.6)	Ref.
Yes	1061 (47.8)	1 (0.84−1.19)	5688 (46)	0.94 (0.87−1.01)	270 (27.1)	1.14 (0.89−1.46)
**Have a stable partner**						
No	1056 (48.2)	Ref.	5403 (44.7)	Ref.	317 (25.5)	Ref.
Yes	391 (47.9)	0.84 (0.71−1)	2456 (47.4)	1.03 (0.96−1.11)	115 (28.2)	1.08 (0.81−1.44)
**How many sexual partners in P6M**						
None			491 (36.4)	Ref.	72 (21.3)	Ref.
1−5			3896 (43.5)	1.12 (0.98−1.28)	256 (27.5)	1.12 (0.79−1.60)
6−10			1450 (49.2)	1.27 (1.09−1.47)	58 (30.1)	1.10 (0.69−1.78)
>10			2002 (50.8)	1.31 (1.13−1.51)	44 (25)	0.96 (0.58−1.59)
**Had condomless receptive anal sex**						
No			3937 (44.9)	Ref.	0 (0)	Ref.
Yes			3900 (46.3)	0.95 (0.89−1.02)	193 (28.2)	1.05 (0.8−1.38)
**When was your last HIV test**						
Past 3 months			3486 (51.4)	Ref.		
3−6 months ago			1408 (44.7)	0.85 (0.78−0.94)		
6−12 months ago			1260 (42.5)	0.82 (0.74−0.9)		
>1 year ago			1705 (39.1)	0.74 (0.68−0.81)		
**Awareness of PrEP**						
No			80 (9.1)	Ref.	22 (7.7)	Ref.
Yes			7779 (47.5)	6.7 (4.71−9.52)	410 (30)	3.94 (2.45−6.34)
**PrEP use**						
Never			5944 (44)	Ref.		
Currently using			1325 (57.6)	1.31 (1.17−1.45)		
**Used in the past**			458 (52.5)	1.27 (1.1−1.46)		
Use of ART^b^						
No	27 (20.6)					
Yes	1417 (49.5)					
**Adherence**						
No	538 (44.4)	Ref.				
Yes	879 (53.1)	1.3 (1.11−1.52)				

Abbreviations: aOR, adjusted odds ratio; ART, antiretroviral therapy; HIV, human immunodeficiency virus; PLHIV, people living with HIV; PrEP, pre‐exposure prophylaxis; P6M, past 6 months.

Bold values significant *p* ≤ 0.05.

^a^ One minimun wage corresponded to BRL 1212 (~USD 242) in 2022.

^b^ Not included in the models due to low numbers.

### 3.4 Predictors of perceiving U = U as completely accurate

For all three study groups, high HIV knowledge increased the odds of perceiving U = U as completely accurate (PLHIV: aOR 1.77 95% CI 1.46−2.15, HIV negative: aOR 1.80 95% CI 1.68−1.94, HIV unknown: aOR 1.77 95% CI 1.36−2.29) (Table 5). Age was also consistently associated with the outcome irrespective of the study group, with older participants (35 years or more) having lower odds of perceiving U = U as completely accurate compared to those aged 30−34 years.

**Table 5:** Factors associated with perceiving the U = U slogan as completely accurate by study group (people living with HIV [PLHIV], HIV negative and HIV unknown), Brazil, 2021 (*N* = 19,513)

	PLHIV (*n* = 2841)		HIV negative (*n* = 15,314)		HIV unknown (*n* = 1358)	
	Completely accurate *N* (%)	aOR (95% CI)	Completely accurate *N* (%)	aOR (95% CI)	Completely accurate *N* (%)	aOR (95% CI)
**Age (years)**						
18−24	229 (86.4)	1.60 (0.96−2.69)	1105 (64.9)	1.05 (0.92−1.21)	216 (52)	1.07 (0.74−1.54)
25−29	595 (86.5)	1.23 (0.85−1.76)	2309 (69)	1.10 (0.98−1.23)	167 (54.9)	0.98 (0.68−1.43)
30−34	829 (86.1)	Ref.	2360 (69.1)	Ref.	129 (56.6)	Ref.
35−44	1483 (81.4)	0.58 (0.44−0.77)	3169 (63.5)	0.80 (0.73−0.88)	109 (40.1)	0.51 (0.35−0.75)
≥45	941 (73.1)	0.32 (0.24−0.43)	1405 (52.2)	0.54 (0.48−0.61)	88 (46.3)	0.73 (0.48−1.11)
**Gender**						
Cisgender man	3997 (81.1)	0.68 (0.32−1.43)	10,125 (64.1)	0.88 (0.69−1.13)	689 (50.5)	1.17 (0.59−2.32)
Other	80 (81.6)	Ref.	223 (63.5)	Ref.	20 (44.4)	Ref.
Sexual orientation						
Gay	3664 (82.3)	1.24 (0.66−2.34)	9069 (66.4)	1.44 (1.16−1.78)	559 (54.6)	1.64 (0.99−2.72)
Bisexual	336 (71.5)	0.60 (0.31−1.19)	1039 (52.2)	0.85 (0.68−1.07)	113 (43.3)	1.15 (0.66−1.98)
Other	77 (75.5)	Ref.	240 (47.9)	Ref.	37 (29.8)	Ref.
Race						
White	2345 (81.5)	Ref.	6237 (64.5)	Ref.	407 (50.7)	Ref.
Pardo	1209 (79.6)	1.17 (0.94−1.46)	2837 (62.3)	0.91 (0.84−0.99)	202 (47.2)	0.97 (0.75−1.27)
Black	499 (83)	1.13 (0.82−1.55)	1228 (67.4)	1.11 (0.99−1.25)	94 (55.3)	1.17 (0.81−1.69)
**Family Monthly Income** ^a^						
<1 minimum wage	479 (73.7)	Ref.	1099 (61.6)	Ref.	106 (48)	Ref.
1−3 minimum wage	1301 (79.8)	1.24 (0.94−1.63)	2971 (63.2)	1.01 (0.89−1.14)	262 (50.7)	1.00 (0.71−1.41)
3−6 minimum wage	1135 (82.6)	1.28 (0.94−1.73)	2853 (65)	1.00 (0.88−1.14)	180 (48.6)	1.01 (0.69−1.47)
>6 minimum wage	1119 (84.8)	1.72 (1.23−2.40)	3294 (65.2)	0.99 (0.87−1.13)	154 (55.6)	1.14 (0.75−1.72)
**Education**						
Up to secondary	1290 (75.5)	Ref.	2717 (61.3)	Ref.	327 (50.4)	Ref.
Tertiary	1550 (82.9)	1.39 (1.10−1.74)	4113 (65)	1.04 (0.95−1.14)	250 (50.5)	0.94 (0.71−1.24)
Post‐tertiary	1227 (85.4)	1.20 (0.91−1.58)	3504 (65.4)	1.08 (0.97−1.20)	131 (50.8)	0.90 (0.63−1.30)
**Live in capital/metropolitan region**						
No	1038 (81.6)	Ref.	2699 (59.8)	Ref.	247 (44)	Ref.
Yes	3039 (81)	0.85 (0.68−1.05)	7649 (65.8)	1.16 (1.07−1.25)	462 (54.5)	1.48 (1.17−1.88)
**Have a stable partner**						
No	2860 (80.7)	Ref.	7269 (64.3)	Ref.	537 (50.8)	Ref.
Yes	1217 (82.2)	0.97 (0.78−1.20)	3079 (63.7)	1.01 (0.94−1.10)	172 (49)	1.02 (0.77−1.36)
**How many sexual partners in P6M**						
None			641 (51.9)	Ref.	125 (41.3)	Ref.
1−5			5140 (62)	1.18 (1.02−1.35)	406 (51.2)	1.28 (0.92−1.78)
6−10			1855 (66.5)	1.23 (1.05−1.44)	100 (61.3)	1.92 (1.20−3.06)
>10			2673 (70.8)	1.37 (1.17−1.60)	71 (51.1)	1.24 (0.77−2.00)
**Had condomless receptive anal sex**						
No			5003 (61)	Ref.	455 (45.5)	Ref.
Yes			5312 (67.4)	1.03 (0.96−1.11)	311 (53.7)	0.98 (0.75−1.27)
**When was your last HIV test**						
<3 months ago			4618 (71.5)	Ref.		
3−6 months ago			1861 (63.3)	0.94 (0.85−1.04)		
6−12 months ago			1643 (59.7)	0.88 (0.79−0.97)		
>1 year ago			2226 (55.8)	0.80 (0.73−0.88)		
**HIV knowledge**						
<12 items correct	1090 (71.5)	Ref.	4769 (56.5)	Ref.	455 (45.5)	Ref.
All items correct	1199 (83)	1.77 (1.46−2.15)	5579 (72.4)	1.80 (1.68−1.94)	254 (62)	1.77 (1.36−2.29)
**Awareness of PrEP**						
No			227 (34.6)	Ref.	58 (28.3)	Ref.
Yes			10,121 (65.4)	2.34 (1.82−3.00)	651 (54.1)	2.17 (1.51−3.13)
**PrEP use**						
Never			7664 (60.7)	Ref.		
Currently using			1882 (83.2)	2.44 (2.14−2.78)		
Used in the past			583 (68.8)	1.24 (1.06−1.45)		
**Use of ART** ^b^						
No	99 (64.3)					
Yes	3973 (81.7)					
**Adherence**						
No	1658 (80.4)	Ref.				
Yes	2316 (82.7)	1.25 (1.03−1.52)				

Abbreviations: aOR, adjusted odds ratio; HIV, human immunodeficiency virus; PLHIV, people living with HIV; PrEP, pre‐exposure prophylaxis; P6M, past 6 months; U = U, undetectable equals untransmittable.

Bold values significant *p* ≤ 0.05.

^a^ One minimun wage corresponded to BRL 1212 (~USD 242) in 2022

^b^ Not included in the models due to low numbers.

For PLHIV, we found that those reporting adherence to ART in the past 7 days had greater odds of perceiving U = U as completely accurate (aOR 1.25 95% CI 1.03−1.52). For HIV‐negative and HIV‐unknown participants, PrEP awareness more than doubled the odds of perceiving U = U as completely accurate (HIV negative: aOR 2.34 95% CI 1.82−3.00, HIV unknown: aOR 2.17 95% CI 1.51−3.13). For the HIV‐negative group, compared to never having used PrEP, those currently using (aOR 2.44 95% 2.14−2.78) and those who had used in the past (aOR 1.24 95% CI 1.06−1.45) had greater odds of perceiving U = U as completely accurate.

## 4 DISCUSSION

Our results showed that the proportion of participants with high HIV knowledge differed by study group, reaching almost half of PLHIV and HIV‐negative participants, and only one‐fourth of HIV‐unknown participants. Prior studies from Brazil that used HIV‐KA (with 12 items) among MSM had found that 24% [18] and 28% [19] of participants had high levels of HIV knowledge in samples from 2016 and 2019 suggesting an increase in HIV knowledge over time. When looking specifically at three items that address PrEP, TasP, PEP, we found that, among all groups, PEP knowledge was highest. Again, as PEP is the oldest of the three interventions, available in Brazil since 1999 through the National Public Health System [30], a slow but progressive dissemination of knowledge about PEP might explain our findings. This hypothesis resonates with the observed frequency of PEP prescriptions in Brazil, which was quite restrictive and slow during the first decade but significantly increased from 2011 onwards as the prescription of PEP after consented sexual exposure became more frequent [30].

We found that accurate perception of the U = U slogan was much higher among PLHIV, reaching 80%, whereas it reached 60% of HIV negative and 43% of HIV‐unknown participants. Conversely, the proportion of individuals reporting “not knowing what undetectable is” was much higher among HIV‐unknown participants. In a prior study conducted in Brazil in 2019, 79% of PLHIV and 44% of HIV‐negative MSM rated the U = U slogan as completely accurate [28]. Similar results were noted in studies conducted among SGM from Australia, the United States and India [26, 31, 32]. It is worrisome that even among healthcare providers, perceived accuracy of the U = U slogan is not pervasive. In a web‐based survey conducted in Brazil (2020), only 74% of physicians who prescribed ART perceived U = U as completely accurate [33]. Reinforcement of the U = U message in HIV treatment and prevention services is urgent to increase U = U literacy among healthcare providers and, per consequence, users [15]. However, it is important to provide a safe space for providers to navigate the health communication complexities and ethics [34] while also promoting research to understand how medical mistrust, HIV stigma and cultural characteristics might factor into the U = U messaging. In this regard, the WHO's policy brief [8] stating in clear and positive language that “zero risk of transmitting HIV is attainable through adherence to appropriate HIV treatment” is a great step towards encouraging healthcare staff's endorsement and promotion of the U = U slogan.

Our exploration of the factors associated with HIV knowledge showed that socio‐economic status, here measured by income and education, was an important predictor of HIV knowledge among all study groups. This association was also reported in two prior analyses of HIV knowledge from Brazil [17, 18]. These results are worrisome as social determinants of health have also been consistently associated with higher HIV incidence, and worse PrEP outcomes, such as adherence, as well as HIV treatment‐related outcomes [35, 36]. Moreover, improving education alone is largely insufficient to succeed in achieving major public health goals. Health education must go beyond improvements in individual knowledge and beliefs. Health literacy, meaning the cognitive and social skills needed to gain access to, understand and effectively use information in ways that foster good health, should be promoted [37]. Additionally, prior work evaluating the acceptability and integration of online health literacy intervention has highlighted the importance of considering cultural characteristics during the design and implementation of interventions for diverse population groups [38].

We found that HIV knowledge, knowledge of biomedical strategies for HIV prevention and accurate perception of the U = U slogan converged. Furthermore, for all study groups, high HIV knowledge was strong and consistently associated with the accurate perception of the U = U slogan even after adjusting for other factors. As one of the items of the HIV‐KA instrument addresses TasP specifically, this may be partially explained by the instruments covering the same ideas. However, it may also be that as one receives information about and understands one aspect of HIV dynamics, then not only does functional literacy increases but also interactive and critical literacy allowing the individual to “extract information and derive meaning from different forms of communication, and to apply new information to changing circumstances” as well as “to critically analyze information, and to use this information to exert greater control over life events and situations” [37]. Importantly, there is evidence to support the link between higher levels of knowledge and actual use of prevention strategies, such as PrEP use [39].

Campaigns can be an effective way to increase HIV knowledge and awareness, particularly among socio‐economically vulnerable groups who may have limited access to health information and services [40, 41, 42]. Moreover, a 2023 meta‐analysis concluded that mass media campaigns can have a modest and context‐specific impact on HIV‐related stigma reduction [43]. In general terms, it is suggested that to be effective campaigns should provide accurate and up‐to‐date information about HIV transmission, prevention, testing and treatment. Additionally, they should be delivered in a way that is culturally appropriate, easy to understand and accessible to different populations (i.e. be disseminated through diverse media channels, such as social media, TV and print media). Moreover, for campaigns targeting specific groups, such as SGM, they should be tailored to address their specific needs and concerns. There have been annual campaigns developed by the Brazilian Ministry of Health aimed at increasing HIV testing and promoting the use of condoms, most frequently released near Carnival [44, 45]. On 1st December 2023, a technical note was released encouraging healthcare providers to incorporate the scientific evidence behind U = U and “zero risk” into HIV care [46]. Broader dissemination among vulnerable populations and beyond are needed, where the use of clear and standard language should be carefully evaluated. The Prevention Access campaign has resources and adaptations of the U = U message that can be tailored to different cultural and linguistic contexts and may be useful (https://preventionaccess.org/resources). Lastly, the impact of campaigns on HIV knowledge and literacy should be evaluated scientifically, in randomized clinical trials.

Our study has limitations to be acknowledged. Although we included a large sample of SGM, we recruited a convenience sample of those who have access to cellphones and use GSN apps or social media thus hindering generalization of the findings to all Brazilian SGM. However, this type of selection bias might be small as recent nationally representative data show that 84% of Brazilians have access to the internet and, among these, 99% access the internet on mobile phones. Our survey was based on self‐reported information including regarding one's HIV status, which we used to create the population groups evaluated in the study.

## 5 CONCLUSIONS

Our findings show that HIV knowledge and perceived accuracy of U = U are strongly correlated and differ according to HIV status among SGM from Brazil. Moreover, higher HIV knowledge was associated with higher income and education regardless of HIV status, and HIV knowledge was a strong predictor of perceived accuracy of the U = U slogan as completely accurate. Educational campaigns regarding PrEP/PEP, TasP and U = U need to focus on achieving health literacy. In line with recent WHO's guidelines, campaigns should contain clear messages that an undetectable viral load means zero risk to increase understanding and use of this information, especially among SGM under social‐economic vulnerability.

## COMPETING INTERESTS

The authors declare no competing interests.

## AUTHORS’ CONTRIBUTIONS

TST and PML conceived and implemented the survey. KROS, TST and PML analysed the data and generated the results. RCF, LEC, VGV and BG provided guidance on results interpretation. KROS, LEC, TST and PML reviewed the literature and drafted the manuscript. All authors critically revised the manuscript for important intellectual content and approved the final version of the manuscript.

## FUNDING

PML was supported by Fundação Oswaldo Cruz: INOVA. TST was supported by the Coordenação de Aperfeiçoamento de Pessoal de Nível Superior (CAPES)—Finance Code 001, CNPq,Fundação Carlos Chagas Filho de Amparo à Pesquisa do Estado do Rio de Janeiro #E‐26/201.133/2021 and #E‐26/201.270/2022 and #E‐26/211.577/2021, Conselho Nacional de Desenvolvimento Científico e Tecnológico and #311871/2021‐6 and #316401/2021‐8 and #402916/2021‐2.

## Supporting information


**Table S1**. Items of the HIV knowledge assessment tool (HIV‐KA).

## Data Availability

A complete de‐identified dataset sufficient to reproduce the study findings will be made available upon request to the corresponding author, following approval of a concept sheet summarizing the analyses to be done.
