# Supplementary material for: Knowledge of HIV transmission, prevention strategies and U = U among adult sexual and gender minorities in Brazil
Source: J Int AIDS Soc. 2024 Feb 20;27(2):e26220. doi: 10.1002/jia2.26220 (PMC10879640; doi:10.1002/jia2.26220)
Supplement: Supplementary file 1 — Table S1. Items of the HIV knowledge assessment tool (HIV‐KA). [file JIA2-27-e26220-s001.docx]

**Supplementary Material**

**Table 1. Items of the HIV knowledge assessment tool (HIV-KA).**

| Item number | Item content |
| --- | --- |
| 1 | There are medications for HIV-negative people to take before having sex with other people to prevent HIV infection. |
| 2 | An HIV-infected person who is taking HIV/AIDS medications has a lower risk of transmitting the virus to another person. |
| 3 | An HIV-infected pregnant woman receiving HIV/AIDS medications during prenatal and at childbirth will have a lower chance of transmitting the virus to the baby. |
| 4 | There are medications for HIV/AIDS to be used after a situation of risk of infection (i.e. unprotected sex, sexual violence, etc). |
| 5 | People can be infected with HIV if they share utensils, cups, or meals. |
| 6 | People can be infected with HIV if they use public toilets. |
| 7 | People can be infected with HIV if it they are bitten by mosquitoes. |
| 8 | When having intercourse with only one faithful partner, not infected with HIV, the risk of contracting the virus is lower. |
| 9 | There is a cure for HIV. |
| 10 | A healthy-looking person may be infected with the HIV virus. |
| 11 | A person can contract HIV if he/she shares with other people instruments for the use of drugs such as syringes, needles etc. |
| 12 | People can contract HIV if they do not use condoms during sexual intercourse. |
